# Neuromodulating attention and mind-wandering processes with multi-session real-time electroencephalogram

**DOI:** 10.1016/j.pbj.0000000000000017

**Published:** 2018-07-16

**Authors:** Óscar F. Gonçalves, Sandra Carvalho, Augusto J. Mendes, Alberto Lema, Jorge Leite, Paulo S. Boggio

**Affiliations:** a Psychological Neuroscience Lab – CIPsi, School of Psychology, University of Minho, Braga, Portugal; b Spaulding Neuromodulation Center, Department of Physical Medicine & Rehabilitation, Spaulding Rehabilitation Hospital and Massachusetts General Hospital, Harvard Medical School, Boston, MA; c Social and Cognitive Neuroscience Laboratory, Center for Health and Biological Sciences, Mackenzie Presbyterian University, São Paulo, Brazil; d Portucalense Institute for Human Development (INPP), Universidade Portucalense, Porto, Portugal.

**Keywords:** attention, mind wandering, neurofeedback, real-time electroencephalogram

## Abstract

Previous studies showed the efficacy of a single session real-time electroencephalogram (rtEEG) protocols in sensorimotor rhythm (SMR) or Theta up-training. However, the impact of this training on sustained or mind-wandering attention was only modest. This could be explained by the lack of specificity in distinct rtEEG training protocols given their limitation in inhibiting or decreasing the amplitude of down-trained bands. Additionally, multiple sessions of rtEEG in up-training/down-training SMR (sensorimotor rhythm) and Theta along with better ways of tracking sustained and mind-wandering attention protocols may be required to achieve consistent effects. Here we describe the effects of a 10-session trial of up-training/down-training SMR or Theta (SMR⇑Theta⇓; Theta⇑SMR⇓), looking at the effects of 2 rtEEG training protocols in 2 n of 1 subject designs. We also tested to impact of this training in sustained and mind-wandering attention during the course of a Sustained Attention Response Task (SART). The present trial confirmed the potentiality of a multi-session protocol in up-training Theta or SMR and, as consequence, increasing sustained attention (Theta up-training) and mind-wandering attention (SMR up-training). However, the simultaneous increase of the Theta amplitude in the SMR⇑Theta⇓ (and the more modest increase of the SMR amplitude in Theta⇑SMR⇓) reduced the specificity of the rtEEG training. Future studies should build on the potentiality of extended rtEEG protocols on this attention paradigm but increasing the specificity of the trained EEG bands choosing less tedious/more motivating feedback instruments (SMR⇑Theta⇓) and conducting the Theta⇑SMR⇓ training eyes closed.

## Introduction

Distinct brain oscillatory rhythms have been associated with different attention processes.^1^ For example, a recent study using the Attention Network Test (ANT) showed that increases in low-frequency band power (theta and alpha) are an electroencephalogram (EEG) marker for the alert and conflict attentional networks, along with a broad power increase for the orientation network. Additionally, the type of interfering thoughts (ie, mind-wandering) during blocks of the ANT did not differentially impact EEG power band in any of the attention networks (alerting, orienting, and conflict).^2^

Studies using real-time EEG (rtEEG) showed that the sensorimotor rhythm (SMR) up-training (13–15 Hz) and Theta down-training (4–8 Hz) significantly impacted attention, as assessed in the Attention Network Task^3^ or Dichotic Listening Task.^4^ However, a previous study by^5^ showed that 2 single sessions of rtEEG protocols were effective in increasing the amplitude of the targeted bands (SMR in SMR⇑Theta⇓; Theta in Theta⇑SMR⇓), but unable to modulate sustained (ie, ANT) and mind-wandering attention.

It is possible that more consistent effects of rtEEG in up-training/down-training SMR and Theta, and the impact of this training on attention processes, require multiple session protocols, along with better ways of tracking sustained and mind-wandering attention. Here we describe the effects of a 2 single subject 10 sessions trial, looking at the effects of 2 rtEEG training protocols (SMR⇑Theta⇓; Theta⇑SMR⇓) in sustained and mind-wandering attention during the course of a Sustained Attention Response Task (SART).

## Method

### Participants

Two healthy college students, 1 female (33 years old) and 1 male (21 years old), with normal or corrected to normal vision participated in the study. Participants provided signed informed consent and the study was approved by the local review board and carried out in accordance with The Code of Ethics of the World Medical Association (Declaration of Helsinki).

### Experimental Procedure

**Figure 1:**
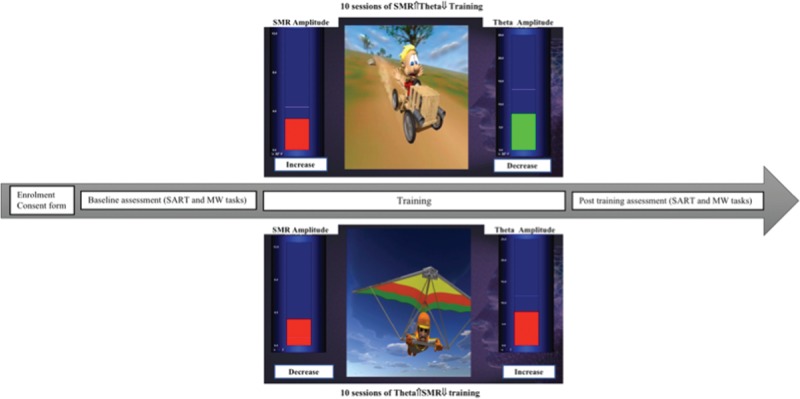
Schematic representation of the study protocol. MW = Mind-Wandering Task, SART = Sustained Attention Response Task, SMR = sensorimotor rhythm.

Participants were randomly assigned to 1 of the 2 rtEEG training protocols: (1) SMR⇑Theta⇓, that is, increase SMR and decrease theta, or (2) Theta⇑SMR⇓, that is, increase theta and decrease SMR. Participants completed the attention (SART) and the MW tasks before and after the rtEEG training (Fig. 1).

Training was conducted with eyes-open with the participants comfortably sat, facing a 15 LED monitor (participant's feedback screen). Silver/silver chloride electrodes were secured using a Nexus Mini-Cap with an EXG cable (attached to EEG disks) and fixed to the skin with the help of 10 to 20 paste and ear clips attached to the ear lobes. A referential montage was used, with the active electrode placed on C_z_ (10–20 system), the reference electrode on the left ear lobe (A_1_) and the ground electrode on the right ear lobe (A_2_). The EEG signals were registered with a 512-Hz sampling rate using a Nexus-32 amplifier with a 24-bit A-D converter (MindMedia, Herten, Netherlands). BioTRace+ software (MindMedia) was used for EEG signal processing and feedback programing. The EEG signal was digitally filtered in order to extract the training bands (SMR, 13–15 Hz; Theta, 4–8 Hz) and artifact control bands (EMG, electromyogram artifact wave; EOG, electroculogram artifact wave). Impedance was kept below 5 Ω. All the data were corrected for artifacts using the Automatic Artifact Rejection option available in BioTRace+, rejecting all amplitudes above 100 μv.

Training consisted of 10 daily session (weekends off) comprising 3′ baseline without feedback, followed by 5 blocks (5′ each) of rtEEG training interleaved with 1′ resting between blocks. One of the participants was instructed to try to increase SMR and decrease Theta amplitudes (SMR⇑Theta⇓protocol) while the other was required to increase Theta and decrease SMR (Theta⇑SMR⇓ protocol). The amplitudes of Theta and SMR waves were presented in a feedback screen by means of bar graphs connected to the SMR and Theta amplitude data channels, respectively. Training thresholds were established calculating the mean amplitude of the training bands in each block (starting with baseline) and establishing the threshold 10% above the mean of the previous block for the up-trained band (SMR for the SMR⇑Theta⇓ protocol; Theta for the Theta⇑SMR⇓ protocol) and 10% below the mean of the previous block for the down-trained band (Theta for the SMR⇑Theta⇓ protocol; SMR for the Theta⇑SMR⇓ protocol). Two types of reinforcement were provided. First, bar graph colors changed from red to green every time participants were able to maintain the signal above or below the threshold (dependent if it was up-training or down-training) for 500 ms. Second, a video animation with a car (SMR⇑Theta⇓ protocol) or a delta-glider (Theta⇑SMR⇓ protocol) would be inhibited (ie, come to a stop) every time participants were not reaching the threshold targets. Additionally, the animation was inhibited, in both protocols, for EMG and EOG amplitudes for more than 500 ms above the established thresholds (10 and 80 μv, respectively).

## Materials

### Sustained attention response task/mind-wandering task

Sustained attention was measured using a go/no go SART task previously developed by Stawarczyk et al.^6^ In this task, a series of numbers (1–9) were presented in the center of a computer screen and participants were asked to respond as quick as possible to all numbers (go trials) with the exception of number 3 (no/go trials) (probability of the no-go stimulus, 11%; interstimulus interval, 2000 ms). The duration of each stimulus was 500 ms and stimuli were presented in blocks (30 in total with variable durations of 25, 35, 45, 55, or 65 seconds). The 5 last stimuli of each block (always go trials) were immediately followed by a thought-probe requiring the participant to identify which type of thoughts were predominant during the preceding block by choosing 1 among the following 4 options: (1) on task (OT)—participant was focused on the task (ie, cues and direction of the arrows); (2) task-related interference (TRI)—participant was focused on side aspects of the task (eg, task duration, concerns about overall performance, rumination over a mistake, etc); (3) external distractions (ED) —participant was focused on stimuli from the current environment but not related to the experimental task, such as overall exteroceptive conditions (eg, light, temperature) or interoceptive conditions (eg, physical sensation, hunger, thirsty, etc); (4) task-unrelated and stimulus-independent experience (SITUT) —the participant wandered through thoughts dissociated either from the task or current exteroceptive or interoceptive conditions (eg, past experience; future plans, etc).

## Results

### The effects of rtEEG in SMR and Theta amplitudes

**Figure 2:**
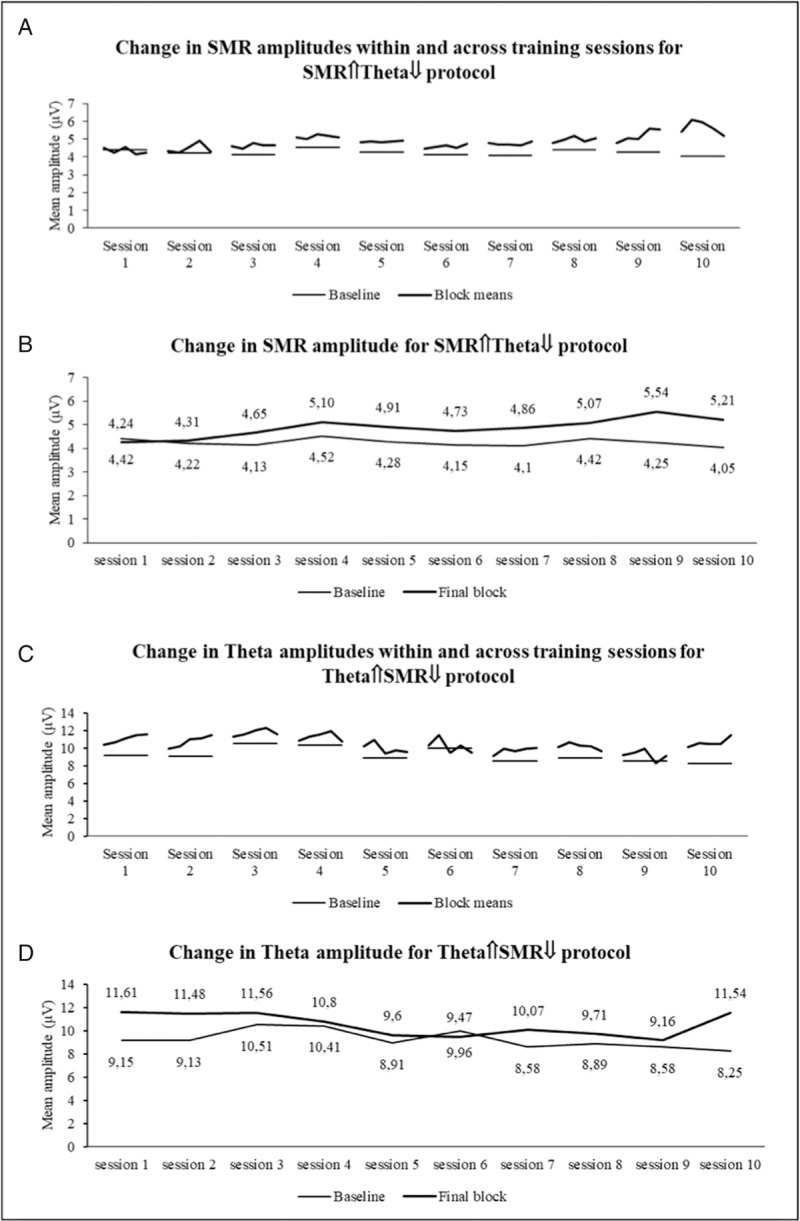
Changes in the uptrained bands—SMR for the SMR⇑Theta⇓ (A and B) and Theta for the Theta⇑SMR⇓ (C and D) protocols. SMR = sensorimotor rhythm.

First, to explore the effects of the 2 rtEEG protocols in modulating the targeted bands (SMR and Theta), we analyzed changes for SMR and Theta mean amplitudes across sessions, and within each session across training blocks for each protocol. As shown in Figure 2A, and confirming the effectiveness of up-training strategies, there was a steady increment across training in the mean SMR amplitude for the SMR⇑Theta⇓ protocol. Figure 2B illustrates the changes in SMR amplitudes for each session between the baseline and the final training block. Overall there was 17.87% increase in the SMR amplitude between the first baseline and the last training block of the 10th session. Likewise, there was an increase in Theta mean amplitude for the Theta⇑SMR⇓ protocol (Fig. 2C). Figure 2D evidences the steady changes in Theta amplitude, within each session, between baseline and the final training block. Also, here there was a 26.12% increase in amplitude between the first baseline and the last session final training block.

**Figure 3:**
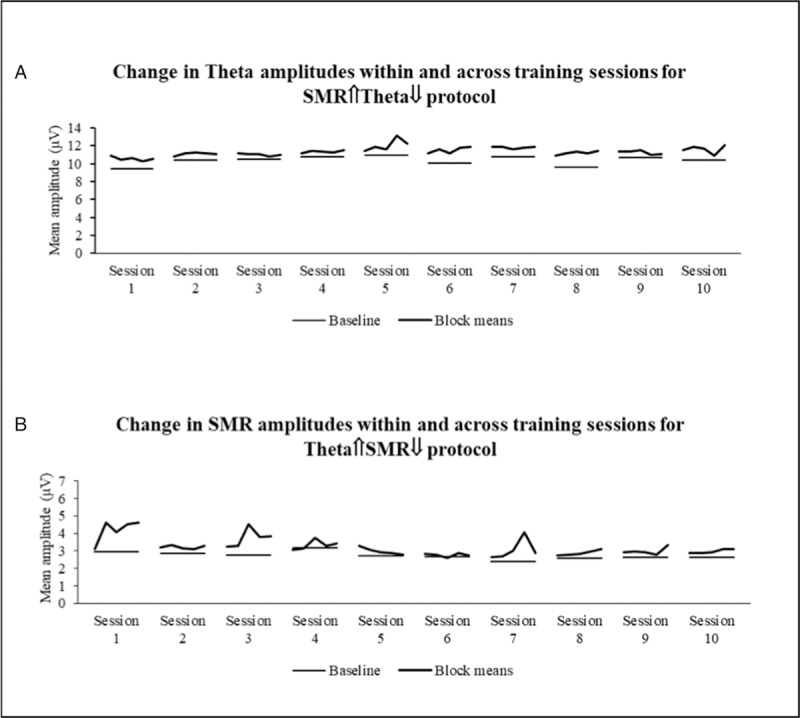
Changes in the downtrained bands—Theta for the SMR⇑Theta⇓ (A), and SMR for the Theta⇑SMR⇓ (B) protocols. SART = Sustained Attention Response Task, SMR = sensorimotor rhythm.

However, as illustrated in Figure 3, none of the protocols was effective in inhibiting the down-trained bands. This was particularly evident in the SMR⇑Theta⇓ protocol in which there was a 27.83% increase in the theta amplitude between initial baseline and final session last training block (Fig. 3A). This increase, even though less dramatic for the Theta⇑SMR⇓ protocol, represented still a 6.12% increase in the SMR amplitude between initial baseline and final session last training block (Fig. 3B).

### Effects of rtEEG on sustained and mind-wandering attention

**Figure 4:**
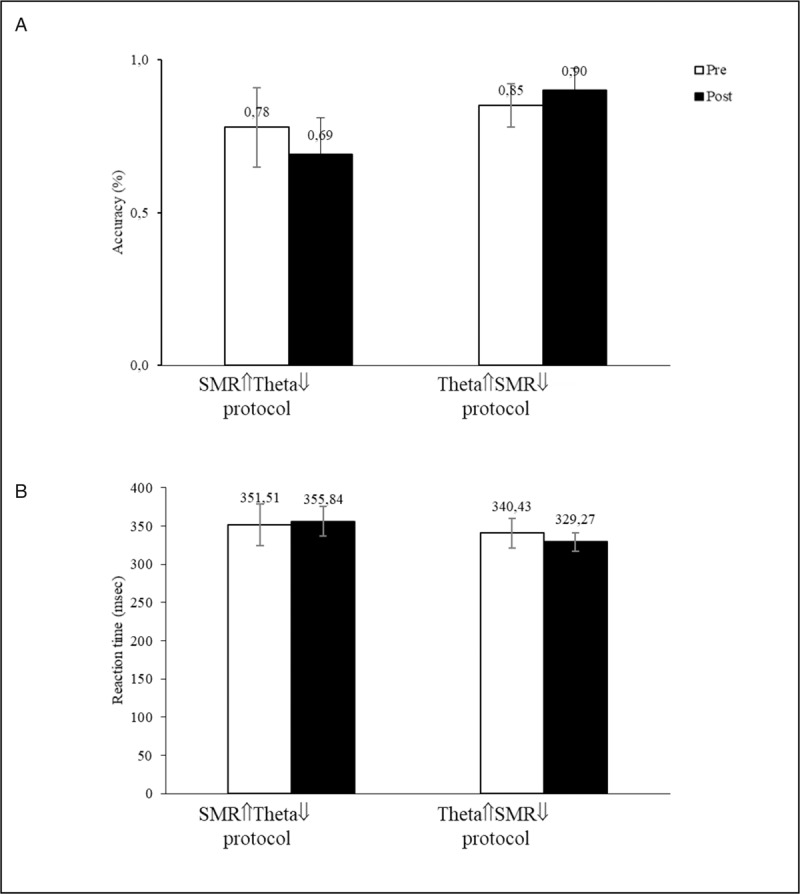
The effects of rtEEG on SART accuracy (A) and reaction time (B). SART = Sustained Attention Response Task.

In terms of sustained attention, and as shown in Figure 4A, the Theta⇑SMR⇓ protocol led to an increase of accuracy contrasting with a decreased accuracy in the SMR⇑Theta⇓ protocol. Consistent with this finding, a decrease in the reaction time was found for Theta⇑SMR⇓ along with a reaction time increase for the SMR⇑Theta⇓ protocol (Fig. 4B)

**Figure 5:**
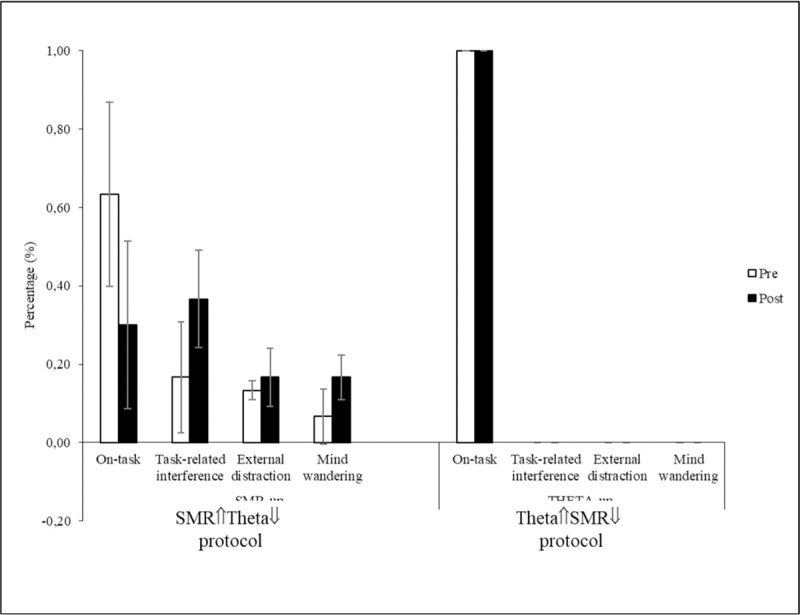
Type of Interfering thoughts during SART before and after rtEEG protocols. SART = Sustained Attention Response Task.

Responses to the thought probe showed that, while in the Theta⇑SMR⇓ the individual remained completely focused on the task both before and after the training, the SMR⇑Theta⇓ was associated with decreased of on-task thoughts (Fig. 5).

### Side effects related with the training

None of the participants reported any side effects during and right after the training sessions.

## Discussion

The objective of this trial was to explore if 10 repeated sessions of 2 neurofeedback protocols were effective in increasing and inhibiting SMR or Theta bands and, consequently, modulate sustained and mind-wandering attention, in healthy volunteers. Overall, confirming previous findings,^5^ both protocols increased the amplitude of the up-trained bands (either SMR or Theta) but failed to decrease the amplitude of down-trained bands, particularly for Theta in the SMR⇑Theta⇓ protocol. A previous study found that this was also the case in a single session protocol. In another study, using a similar protocol, we confirmed the difficulty in selectively inhibiting Theta in 1 single session training protocol.^5^ The objective of the current trial was to test if we could possibly reverse this effect in a multiple sessions training as suggested by other studies.^7^ Apparently this was not the case. Our participant reported to have almost double the degree of sleepiness as a consequence of the SMR⇑Theta⇓ protocol and this may have contributed to the simultaneously increase of theta amplitude. The feedback instrument used in this protocol (ie, moving car) may be too tedious and end up prompting low frequency oscillatory rhythms. Also, the limited efficacy in inhibiting the SMR amplitude in the Theta⇑SMR⇓ may be explained by the fact that the training was conducted with eyes open and as such associated with a level of vigilance, thus requiring significant SMR amplitude.

An increased in sustained attention (both in terms of accuracy and reaction) was associated with the Theta⇑SMR⇓ contrasting with a decrease for the SMR⇑Theta⇓ training. Apparently, with a more extended training (10 sessions) and using a distinct more focused attention measure, we found initial indication for the effect of training increased Theta on sustained attention. This is a very promising result, consistent with the finding that at least certain sustained attention tasks (e.g., alert, conflict) were associated with the predominance of low frequency bands, particularly Theta. Consistently, responses to the thought probes indicate that in the Theta⇑SMR⇓ protocol the individual remained focused on the task between pre and post-training while the SMR⇑Theta⇓ was associated with an increase of mind-wandering thoughts.

Summing up, the present trial confirmed the potentiality of a multi-session protocol in up-training Theta or SMR and as consequence, increasing sustained attention (Theta up-training) and mind-wandering attention (SMR up-training). However, the simultaneously increase of the Theta amplitude in the SMR⇑Theta⇓ (and the more modest increase of the SMR amplitude in Theta⇑SMR⇓) reduced the specificity of the rtEEG training introducing a possible confounding effect. Future studies should build on the potentiality of extended rtEEG protocols on this attention paradigm but increasing the specificity of the trained EEG bands choosing less tedious/more motivating feedback instruments (SMR⇑Theta⇓) and conducting the Theta⇑SMR⇓ training eyes closed.

## Acknowledgments

None.

## Funding

ÓFG was funded by the Brazilian National Counsel for Scientific and Technological Development (CNPq) as a special visiting researcher (Grant 401143/2014-7). This study was partially conducted at the Psychology Research Centre (UID/PSI/01662/2013), University of Minho, and supported by the Portuguese Foundation for Science and Technology and co-financed by FEDER through COMPETE2020 under the PT2020 (grant POCI-01-0145-FEDER-007653). Paulo S. Boggio is a CNPq researcher fellow (grant 311641/2015-6). SC is funded through the Portuguese Foundation for Science and Technology (IF/00091/2015). JL is funded through the Portuguese Foundation for Science and Technology (PTDC/MHC-PCN/3950/2014).

## Ethical approval

All participants provided signed informed consent and the study was approved by the local review board and carried out in accordance with The Code of Ethics of the World Medical Association (Declaration of Helsinki).

## Conflicts of interest

The authors report no conflict of interests.

## References

[1] Braboszcz C Delorme A Lost in thoughts: neural markers of low alertness during mind wandering. *Neuroimage* 2011; 54:3040–3047. 20946963 10.1016/j.neuroimage.2010.10.008

[2] Gonçalves OF Rêgo GG Conde T Mind wandering and task-focused attention: ERP correlates. *Scientific Rep* 2018; 8:7608. 10.1038/s41598-018-26028-w PMC5953943 29765144

[3] Hill A Barnea A Herzberg K Aboitiz F Cosmelli D Measuring and modulating hemispheric attention. *From Attention to Goal-Directed Behavior: Neurodynamical, Methodological and Clinical Trends*. Berlin, Heidelberg:Springer; 2009 125–143.

[4] Gadea M Aliño M Garijo E Testing the benefits of neurofeedback on selective attention measured through dichotic listening. *Appl Psychophysiol Biofeedback* 2016; 41:157–164. 26683198 10.1007/s10484-015-9323-8

[5] Gonçalves ÓF Carvalho S Mendes A Neuromodulating attention and mind-wandering processes with a single session real time EEG. Appl Psychophys Biof 2018. 10.1007/s10484-018-9394-4 29797155

[6] Stawarczyk D Majerus S Maj M Mind-wandering: Phenomenology and function as assessed with a novel experience sampling method. *Acta Psychol (Amst)* 2011; 136:370–381. 21349473 10.1016/j.actpsy.2011.01.002

[7] Vernon D Egner T Cooper N The effect of training distinct neurofeedback protocols on aspects of cognitive performance. *Int J Psychophysiol* 2003; 47:75–85. 12543448 10.1016/s0167-8760(02)00091-0

